# Editorial: The heart lung disease: a need for novel definitions and understanding of pathological overlaps in the COVID-19 era and beyond

**DOI:** 10.3389/fcvm.2023.1329841

**Published:** 2023-12-14

**Authors:** Alaa Mabrouk Salem Omar, Sridhar Chilimuri, Jonathan N. Bella

**Affiliations:** ^1^ Department of Cardiology, Mount Sinai Morningside, New York, NY, United States; ^2^ Icahn School of Medicine at Mount Sinai, New York, NY, United States; ^3^ Department of Cardiology, BronxCare Hospital Center, Bronx, NY, United States; ^4^ Department of Medicine, BronxCare Hospital System, Bronx, NY, United States

**Keywords:** heart lung disease, cardiopulmonary interaction, pulmonary hypertension, COVID-19, angiotensin-converting enzyme (ACE)

**Editorial on the Research Topic** The heart lung disease: a need for novel definitions and understanding of pathological overlaps in the COVID-19 era and beyond

“When your mind tries to verify a preconceived notion, you can miss the obvious.”

**James Cook**

17 November 1728–14 February 1779

Cardiopulmonary interactions reveal themselves in health and disease. Physiological, cardiac, and pulmonary functions are inseparably intertwined by tightly regulated neuro-chemical changes in heart and respiratory rates, changes of intrapleural and intracardiac pressures, mechanical ventricular interdependence, and parallelly arranged pre-capillary low-pressure (pulmonary/right heart) and post-capillary high-pressure (left heart/systemic circuit) system (1–3). The outcome of such cardiopulmonary coupling (CPC) is the functional continuity of the ventilation/perfusion relationships. The disruption of CPC mechanisms at any level would exhibit a significant overlap in clinical, pathophysiologic, and management strategies. Such overlap would manifest in almost all common acute and chronic cardiac and pulmonary conditions and is referred to as “heart–lung disease (HLD).”

One famous example of such overlap is pulmonary hypertension, a disease that in the vast majority of cases is caused by a cardiac condition but can also be caused by pulmonary conditions such as obstructive airway diseases and other conditions such as idiopathic pulmonary vascular remodeling, thromboembolism, and other systemic diseases (2). The result is a disease that has bidirectional etiological and pathological effects on both the cardiac mechanical and hemodynamic functions and the pulmonary vascular properties and ventilatory functions. With both its pre-capillary and post-capillary components, the management of this disease involves toggling between pulmonologists and cardiologists for workup and management. It is not until recently that focused pulmonary hypertension interdisciplinary management groups were constructed and, in reality, such groups exclusively deal with the paradigm of CPC and HLD.

The understanding of CPC as an anatomical and functional interdependence has continued to evolve for the past 2000 years. The accumulating knowledge suggests that a pulmonary or a cardiac pathology seldom exists without an effect on the counter-organ (2). In other words, if you see pulmonary manifestations of any disease, always look at the heart and see how it is also affected and vice versa. Despite the accumulating knowledge, awareness of the nature, extent, and clinical value of HLD among cardiologists and pulmonologists remain largely underexplored, and to this day, efforts in managing the overlaps that exist between pulmonary and cardiac pathologies seem largely separated working efforts among both worlds calling for a need to increase the awareness among clinicians. More interestingly, HLD can manifest itself in situations when it is least expected, many systemic and autoimmune diseases can indirectly affect either the heart or the lung, and through the aforementioned functional and anatomic continuity, the other organ usually follows as a full-blown HLD.

In this issue, the basics of CPC mechanisms and relationships and pathological manifestations as “HLD” have been eloquently summarized by Ronderos Botero et al. Furthermore, using the discussed basic concepts, they shed light on the pathophysiology of the cardiopulmonary disruption on an even more under-discovered scope of HLD in the pathophysiology of myopathies. In this article, myopathy of any sort can cause cardiac and pulmonary pathologies that can result either as a consequence of a co-existing cardiopulmonary structural problem or just as a result of myopathy-related physiological alterations. In-depth discussion of such a less known condition in the clinical case presented served as another indicator of the underestimated cardiopulmonary continuity in diseases when it is least expected or diseases that are expected to affect one organ more than the other.

In another article in this issue, the clinical effects of a presumed cardiopulmonary disruption were reported by Zheng et al., who studied the long-term effect of the co-morbidity of chronic obstructive pulmonary disease (COPD) among Chinese patients with ischemic coronary artery disease treated with percutaneous coronary intervention (PCI) (Zheng et al.). While they have shown an overall lack of association with major cardiovascular outcomes, it is still interesting to note as they have reported a bimodal response where the outcomes become more prevalent in COPD patients in a late stage of follow-up after PCI. Interestingly, the analysis also suggests a complex multifactorial interaction in this group of patients where the outcomes seem to be more prevalent in the two extremes of the age groups and in the presence of specific co-morbidities such as renal dysfunction and peripheral vascular disease that were more prevalent in COPD before propensity score matching.

Admittedly, while there is a large unfamiliarity with the extent of CPC and their pathological impact on HLD, the COVID-19 pandemic was perhaps an eye-opener and a reminder for all of us that we need to dive to deeply understand these complex mechanisms (4, 5). What was initially recognized as a disease that primarily affects the lungs turned out to be a systemic disease that impacts the heart and the lungs in a very close manner suggesting that COVID-19 follows the outlines of the proposed paradigm of HLD. Reportedly, mechanisms involved with local and systemic “inflammation-induced thrombosis” and dysfunction in the renin–angiotensin system and the angiotensin-converting enzyme and its receptors have been proposed as mechanisms to explain HLD in COVID-19 patients through systemic effects as well as local endothelial dysfunction results (6). In this issue, Liu et al. revisited the literature for molecular mechanisms of endothelial dysfunction and thrombo-inflammation, while Camargo et al. studied the role of the renin–angiotensin system in severe COVID-19 and suggested angiotensin II during admission as a predictor of mortality and outcomes of the disease through an analysis in patients from the “Safety and Outcomes Associated with the Pharmacological Inhibition of the Kinin–Kallikrein System in Severe COVID-19.” Despite that large scientific gaps remain in the pathogenesis of COVID-19, a large literature that suggests the relationship of these inflammations to pulmonary and cardiac involvement in patients with COVID-19 is found, and they seem to be largely interrelated. Furthermore, the impact of previous and forthcoming variant waves may compound our understanding of the pathophysiology of CPC in these patients (5).

In conclusion, the unfamiliarity with cardiopulmonary interaction has become unacceptable today, especially since its presence in a newly identified disease such as COVID-19 has surprised us in a way that greatly impacted our ability to predict and understand the course of the disease especially in its initial phases. However, in addition to the great myriad of pathologies that can disrupt the cardiopulmonary coupling as a result of mechanical insults, ischemia, and circulating vasoactive, prothrombotic, inflammatory chemicals, the fact that COVID-19 is not the last respiratory virus with concomitant lethal cardiac effects brings HLD to the forefront of our attention as clinicians calling for more unified efforts both at the bench and the bedside toward creating clear management protocols for which further studies are required.
